# *Veronaeaaquatica* sp. nov. (Herpotrichiellaceae, Chaetothyriales, Eurotiomycetes) from submerged bamboo in China

**DOI:** 10.3897/BDJ.9.e64505

**Published:** 2021-09-23

**Authors:** Sajini K.U. Chandrasiri, Yu-lin Liu, Jun-En Huang, Milan C. Samarakoon, Saranyaphat Boonmee, Mark S. Calabon, Dian-Ming Hu

**Affiliations:** 1 College of Bioscience and Bioengineering, Jiangxi Agricultural University, Nanchang 330045, China College of Bioscience and Bioengineering, Jiangxi Agricultural University Nanchang 330045 China; 2 Jiangxi Environmental Engineering Vocational College, Ganzhou 341002, China Jiangxi Environmental Engineering Vocational College Ganzhou 341002 China; 3 Center of Excellence in Fungal Research, Mae Fah Luang University, Chiang Rai 57100, Thailand Center of Excellence in Fungal Research, Mae Fah Luang University Chiang Rai 57100 Thailand; 4 School of Science, Mae Fah Luang University, Chiang Rai 57100, Thailand School of Science, Mae Fah Luang University Chiang Rai 57100 Thailand; 5 Bioengineering and Technological Research Centre for Edible and Medicinal Fungi, Jiangxi Agricultural University, Nanchang 330045, China Bioengineering and Technological Research Centre for Edible and Medicinal Fungi, Jiangxi Agricultural University Nanchang 330045 China

**Keywords:** one new taxon, hyphomycetes, molecular phylogeny, saprobe, taxonomy, freshwater fungi

## Abstract

**Background:**

Freshwater fungi are highly diverse and ecologically important in freshwater systems. In China, more than 1000 species of freshwater fungi are known. Here, we present a brown-spored hyphomycetes that was collected on a submerged decaying bamboo culm in a forest stream in China.

**New information:**

Phylogenetic analyses of combined LSU, ITS and TUB2 sequences confirm the placement of our new strain in *Veronaea* (Herpotrichiellaceae), sister to *V.japonica*. *Veronaeaaquatica* sp. nov. differs from related taxa *V.compacta* and *V.japonica* in having longer conidiophores and cylindrical to pyriform or subclavate conidia with 0–2 septa. *Veronaeaaquatica* also has darker brown hyphae compared to *V.japonica*. A morphological description and detailed illustrations of *V.aquatica* are provided.

## Introduction

Freshwater fungi are those taxa that grow in freshwater bodies for the entirety or only part of their life cycle (Goh and Hyde 1996, Dhanasekaran et al. 2006). They are "recyclers" in that they decompose dead organic matter (Harms et al. 2011, Iskandar et al. 2011, Anastasi et al. 2013, Tsui et al. 2016, Grossart et al. 2019, Gulis et al. 2019). Freshwater fungi can be found in living (plants and animals) and non-living (decaying wood and leaves) substrates (Choi et al. 2019, Grossart et al. 2019, Tsui et al. 2000). They are accommodated in eight phyla: Aphelidiomycota, Ascomycota, Basidiomycota, Blastocladiomycota, Chytridiomycota, Monoblepharomycota, Mortierellomycota and Rozellomycota (Zhang et al. 2012, Bao et al. 2019, Calabon et al. 2020, Hyde et al. 2021). The majority of described freshwater fungi are members of Dothideomycetes and Sordariomycetes; however, several taxa have been recorded from Eurotiomycetes (Liu et al. 2015, Luo et al. 2016, Luo et al. 2018b, Luo et al. 2019, Dong et al. 2018, Dong et al. 2020a, Wang et al. 2019).

*Coreomyceschinensis* and *C.minor* (Laboulbeniaceae, Laboulbeniales, Laboulbeniomycetes) were the first freshwater taxa reported from China (Thaxter 1931, Hu et al. 2013). During the past two decades, studies have used combined morphological and molecular data to describe freshwater taxa from China (Tsui et al. 2000, Li et al. 2017, Luo et al. 2017, Luo et al. 2018a, Luo et al. 2018b, Luo et al. 2019, Su et al. 2018, Bao et al. 2020, Lu et al. 2020). Hu et al. (2013) reviewed the biodiversity of freshwater fungi in China and reported 782 species. Since Hu et al. (2013), this number has increased to more than 1,000 (Luo et al. 2019, Bao et al. 2020, Dong et al. 2020a, Dong et al. 2020b).

*Veronaea* (Herpotrichiellaceae, Chaetothyriales) was introduced by Cifferi and Montemartini (1958) and is typified by *V.botryosa*, which was isolated from a decomposed rachis of a palm (Arecaceae) in Italy (Arzanlou et al. 2007). Twenty species have been described in *Veronaea*, but sequences are only available for four of them (*V.botryosa*, *V.compacta*, *V.constricta* and *V.japonica*) (Wijayawardene et al. 2020). Only the asexual morphs of *Veronaea* are presently known and they are related to black yeasts (Vicente et al. 2008, Badali et al. 2013, Bonifaz et al. 2013, Döğen et al. 2013).

Species of *Veronaea* are characterised by polyblastic, terminally integrated, cylindrical, solitary, pale brown conidiogenous cells and smooth-walled, septate, cylindrical to pyriform pale brown to brown conidia (Arzanlou et al. 2007, Vicente et al. 2008, Badali et al. 2013, Döğen et al. 2013). There are three records of *Veronaea* species from freshwater habitats, all on submerged wood. These are *V.botryosa* from Thailand *(Dong et al. 2018*), *V.coprophila* from the Republic of Seychelles (Hyde and Goh 1998) and *V.oblongispora* from Hong Kong (Tsui 1999). Since most *Veronaea* species lack molecular data, recollecting and sequencing are essential to investigate the phylogenetic relationships amongst species.

In the present study, we introduce *Veronaeaaquatica* sp. nov., a freshwater species from submerged decaying bamboo culms collected in a stream in Jiangxi Province, China. A morphological description, illustrations and a multi-loci phylogeny are presented. The new species is compared with related taxa.

## Materials and methods

### Sample collection and morphological examination

Submerged decaying bamboo culms were collected from a small forest stream in Lushan Mountains, Jiangxi Province, China in December 2017. Samples were incubated at room temperature for two weeks. Microscopic observation was conducted following Hu et al. (2010) and fungal characters were documented using a microscope. The holotype and ex-type living culture were deposited in the Herbarium of Fungi, Jiangxi Agricultural University (HFJAU), Nanchang-China and Jiangxi Agricultural University Culture Collection (JAUCC), respectively.

### Fungal isolation

Single conidia were isolated in the potato dextrose agar (PDA) plate, following the method of Zhang et al. (2013). Germinated conidia were transferred to PDA plates and incubated at 16°C. Colonial characteristics were described after obtaining the pure cultures.

### DNA extraction and PCR amplification

Mycelia were scraped off from six week-old colonies grown on PDA and transferred into a 1.5 ml centrifuge tube, followed by grinding in liquid nitrogen. DNA was extracted from the ground mycelium using the EZ gene TM fungal gDNA kit (GD2416) according to the manufacturer’s instructions. The partial large subunit rDNA (LSU), internal transcribed spacer (ITS) and partial beta-tubulin (TUB2) were amplified using primer pairs LR0R/LR5, ITS1/ITS4 and T1/Bt2b, respectively (Vilgalys and Hester 1990, White et al. 1990, Hopple 1994, Glass and Donaldson 1995, Rehner and Samuels 1995, O'Donnell and Cigelnik 1997). The amplifications were performed according to Hu et al. (2012) as follows: initial denaturation at 94°C for 3 minutes; followed by 35 cycles of denaturation at 94°C for 30 seconds, annealing at 56°C for 50 seconds, elongation at 72°C for 1 minute; and a final extension at 72°C for 10 minutes. Purification of PCR products and sequencing, using the same primers, were outsourced to Tsingke Biological Technology Company (Beijing, China).

### Sequencing and sequence alignment

Consensus sequences were obtained using Lasergene SeqMan Pro v. 7. BLASTn searches were performed to identify highly similar sequences in NCBI GenBank. Other sequences, used in the phylogenetic analyses (Table 1), were downloaded from NCBI GenBank, based on recently-published data (Dong et al. 2018, Wijayawardene et al. 2020). Single-locus alignments were generated with MAFFT v. 7.036 (http://mafft.cbrc.jp/alignment/server; Katoh et al. 2019). Alignments were further improved manually when necessary in BioEdit v. 7.0.5.2 (Hall 1999). Ambiguous bases were removed using TrimAl v. 1.3 and the gappyout option (Capella-Gutiérrez et al. 2009).

### Phylogenetic analysis

Phylogenetic analyses were performed for both individual (LSU, ITS, TUB2) and combined (LSU-ITS-TUB2) datasets. Maximum Likelihood (ML) analyses were performed in the CIPRES Science Gateway v. 3.3 using the RAxML-HPC2 on XSEDE tool (Stamatakis et al. 2008, Miller et al. 2010, Stamatakis 2014). For each single-locus sequence alignment, GTRGAMMA + I was selected as the best-fit model in MrModeltest 2.3 (Nylander 2004). Bayesian (BYPP) analysis was performed using MrBayes v. 3.1.2. for the combined dataset (Ronquist and Huelsenbeck 2003). Six simultaneous Markov Chains were run for 2,000,000 generations and trees were sampled every 100th generation. The first 2,000 trees were discarded as burn-in; the remaining 18,000 trees were used for calculating posterior probabilities (PP) (Cai et al. 2006). Phylograms were visualised using FigTree v. 1.4.0 (http://tree.bio.ed.ac.uk/software/figtree/). Modification of the final phylogenetic tree was done in Microsoft PowerPoint.

## Taxon treatments

### Veronaea aquatica

Chandrasiri, J.E. Huang & D.M. Hu sp. nov.

6FE5DF25-4037-5B71-8449-5C6134814F7F

IF558295

FoF 05435

#### Materials

- **Type status:**Holotype. **Occurrence:** recordedBy: J.E. Huang; **Taxon:** kingdom: Fungi; phylum: Ascomycota; class: Eurotiomycetes; order: Chaetothyriales; family: Herpotrichiellaceae; genus: Veronaea; specificEpithet: *aquatica*; taxonRank: species; **Location:** country: China; stateProvince: Jiangxi Province; locality: Lushan Mountains; verbatimElevation: 675; verbatimLatitude: 29°55'72''N; verbatimLongitude: 115°94'86''E; **Identification:** identifiedBy: Sajini K. U. Chandrasiri; **Event:** year: 2017; month: December; day: 31; habitat: stream in small forest, on submerged decaying bamboo culms; fieldNotes: Freshwater; **Record Level:** institutionID: HFJAU 0739; institutionCode: Herbarium of Fungi, Jiangxi Agricultural University; collectionCode: HJ054
- **Type status:**Other material. **Record Level:** type: ex-type living culture; collectionID: JAUCC2549; collectionCode: Jiangxi Agricultural University Culture Collection

#### Description

Saprobic on submerged decaying bamboo (Fig. 2). **Sexual morph**: Undetermined. **Asexual morph**: Hyphomycetous. Colonies effuse, spreading very widely, brown to dark brown, white hairy. Mycelium in the wood immersed or partly superficial, hyphae subhyaline to pale olivaceous, smooth, 1.5–3 μm wide. Conidiophores erect, the lower part is usually straight and the upper half is usually flexuose, usually loosely branched, macronematous, monomenatous, sometimes geniculate, smooth-walled, near the apex pale brown, dark brown at the middle and base, 2.5–4 μm wide and up to 280 μm long. Conidiogenous cells terminally integrated, polyblastic, occasionally intercalary, cylindrical, (3–)10–30 × 2–3.5 µm (x̄ = 16.5 × 2.5 μm, n = 30), variable in length, pale brown, later often becoming septate, fertile part subhyaline, wide at the basal part, rachis with crowded, flat to slightly prominent, faintly pigmented; scars flat, slightly pigmented, not thickened, about 0.65 μm diam. Conidia solitary, smooth, cylindrical to subpyriform and some subclavate, 6–11(–12) × 2.5–3.5(–4.0) µm (x̄ = 8.7 × 3.1 μm, n = 50), pale brown, most medially 1-spetate, rarely 0 or 2-septate, often constricted at the septum and the colour septum middle brown and the conidia with a round apex and truncate base; with a faintly darkened, unthickened hilum, about 0.5–0.9 μm diam.

**Culture characteristics**: Conidia germinating on PDA within 24 hrs. Colonies growing on PDA, circular, reaching 10–20 mm diam. after 2–3 weeks at 28°C, *from above* flat, dense, olivaceous to medium brown, lightly raised at centre, surface rough; *from below* medium to dark brown.

##### Etymology

Referring to the aquatic habitat.

#### Notes

*Veronaeaaquatica* is morphologically most similar to *V.japonica* and *V.botryosa*. However, *V.aquatica* has 0–2-septate conidia, whereas those of *V.japonica* are 0–1-septate. In addition, the conidiophores of both *V.compacta* (up to 50 μm) and *V.japonica* (up to 65 μm) are shorter compared to *V.aquatica* (280 μm). *Veronaeaaquatica* has conidiogenous cells that are 10–30 μm in length, while those of *V.botryosa* are 100 μm long. In addition, the conidiophores of *V.aquatica* are 280 μm long; they are shorter in *V.botryosa* (73–124 μm) (Fig. 2).

**Figure 1.**
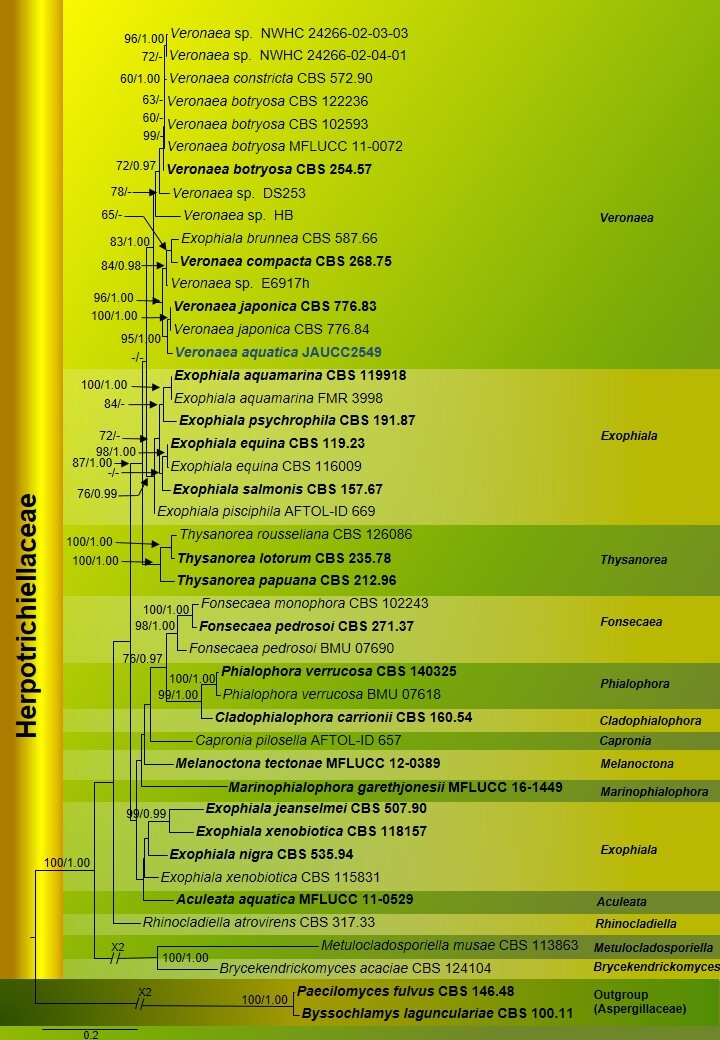
Phylogenetic tree generated from RAxML analysis of a combined ITS, LSU and TUB2 dataset. ML bootstrap (BS) support values ≥ 60% and Bayesian PP ≥ 0.95 are indicated above branches as MLBS/PP. *Paecilomycesfulvus* (CBS 146.48) and *Byssochlamyslagunculariae* (CBS 100.11) serve as outgroup taxa. Type strains are highlighted in bold; the new species is shown in blue bold.

**Figure 2.**
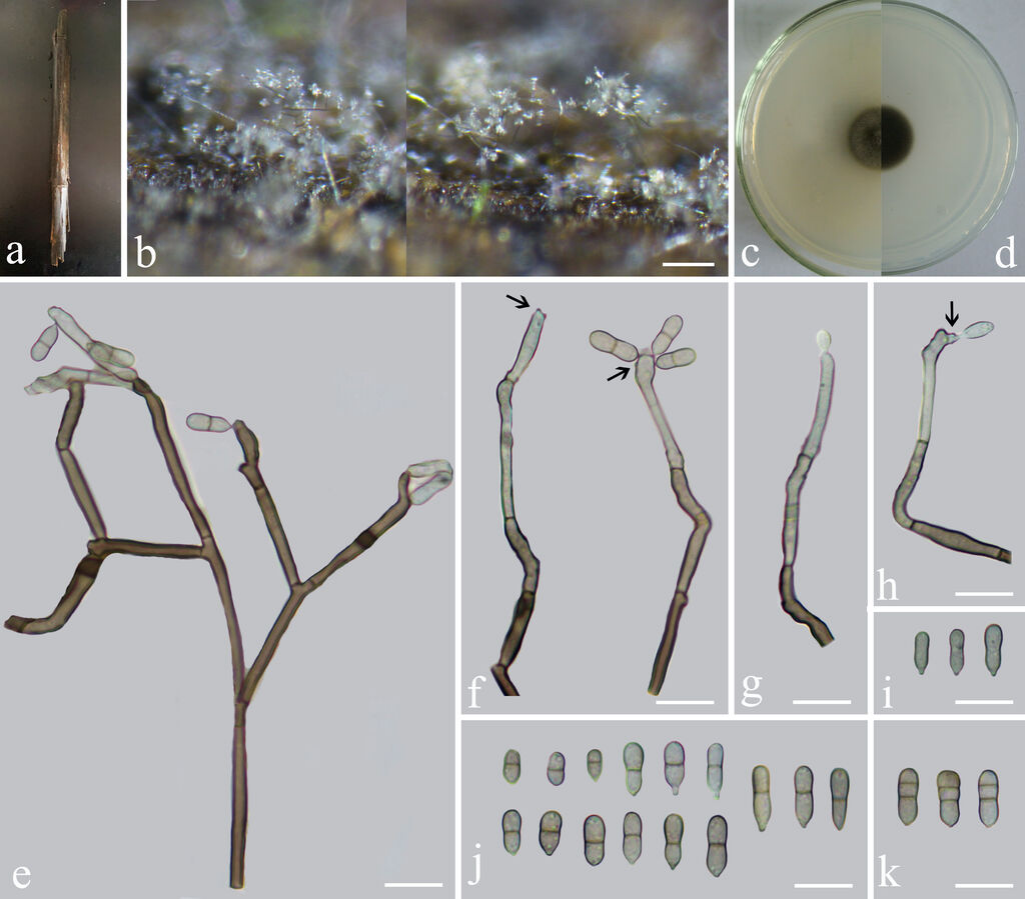
***Veronaeaaquatica*** (HFJAU0739, holotype) **a** Submerged bamboo; **b** Colonies on submerged bamboo culm; **c, d** Colony on PDA from above and below; **e–h** Conidiophores, conidiogenous cells and conidia; note the scars (arrowed in e, d); **i–k** Conidia. Scale bars: b = 100 μm, e–k = 10 μm.

*Veronaeaaquatica* shares the highest identity with *V.japonica* (CBS 776.83) in its LSU (99.65%) and ITS (98.08%). In its TUB2, it shares 89.61% identity with *Exophialabrunnea* (CBS 587.66). However, not enough TUB2 data are available to make conclusions about relationships, based on this gene region. Our tree topology (Fig. 1) is similar to Wang et al. (2019), although these authors did not include *E.brunnea* (CBS 587.66) in their analysis. In our study, *E.brunnea* (CBS 587.66) is clustered with *V.compacta* (CBS 268.75) with poor support (Fig. 1).

## Analysis

### Phylogenetic analyses

The final aligned concatenated dataset (LSU, ITS, TUB2) was comprised of 44 strains including two outgroup taxa, *Byssochlamyslagunculariae* (CBS 100.11) and *Paecilomycesfulvus* (CBS 146.48) (Aspergillaceae) and 734 distinct alignment patterns, with 23.30% of undetermined characters or gaps. The best-scoring RAxML tree (-lnL = 12666.921) is shown in Fig. 1. Tree topologies from ML and Bayesian analyses were congruent; no significant differences were observed at the generic level. *Veronaeaaquatica* (JAUCC2549) was retrieved as sister to *V.japonica* with high support (MLBS = 95%, PP = 1.00).

## Discussion

The family Herpotrichiellaceae (Eurotiomycetes) was introduced by Munk (1953) and currently includes 16 genera (Wijayawardene et al. 2020). The anamorph–teleomorph relationships within Herpotrichiellaceae were described by Müller et al. (1987) and Untereiner et al. (1995). Most anamorphs are dematiaceous and opportunistic fungi (*Capronia*, *Cladophialophora*, *Exophiala*, *Veronaea*) (Untereiner et al. 1995, Crous et al. 2007).

Species of *Veronaea* can be found on wood submerged in freshwater, in soil and on different terrestrial hosts. Fungi in the genus are saprobes (*V.coprophila*, *V.japonica*) or pathogens of plants (*V.ficina*, *V.filicina*) (Kharwar and Singh 2004, Arzanlou et al. 2007, Dong et al. 2018). *Veronaeabotryosa* is a human pathogen, which causes phaeohyphomycosis disease (Kondo et al. 2007, Sang et al. 2011, Bonifaz et al. 2013). *Veronaea* is widely distributed across Australia, Brazil, China, Egypt, India, New Zealand, North America, South Africa and the UK (Dingley 1972, Papendrof 1976, Morgan-Jones 1982, Moustafa and Abdul-Wahid 1990, Kharwar and Singh 2004, Soares and Barreto 2008, Pan et al. 2009, Pan et al. 2012, Pan and Zhang 2010).

This paper introduces a new species of *Veronaea*, bringing the number of species to twenty-one, based on morphology and molecular phylogenetic analyses. We compared the new species to the most related species in Table 2. Several unidentified *Veronaea* species have also been isolated, such as *Veronaea* sp. DS253 (from root of *Boutelouadactyloides*), *Veronaea* sp. E6917h (from *Socrateaexorrhiza*), *Veronaea* sp. HB (from grapevine), *Veronaea* sp. [NWHC 24266–02–03–03; NWHC 24266–02–04–01 (from snake)] (Fig. 1). These taxa are needed to be studied and identified in future research. The Kingdom of Fungi is an incredibly diverse group, with many taxa awaiting discoveries— including those from freshwater habitats. Exploring new fungal taxa, understanding their ecology and generating molecular phylogenetic data will promote fungal conservation (Cheek et al. 2020).

## Supplementary Material

XML Treatment for Veronaea aquatica

## Figures and Tables

**Table 1.** Table of taxa used in this study and GenBank accession numbers of DNA sequences. The new strain is indicated in bold and type strains are indicated with an asterisk ^(*)^.

**Name**	**Strain Number**	**Gene bank accession number**		
		**LSU**	**ITS**	**TUB2**
Aculeata aquatica ^*^	MFLUCC 11–0529	MG922575	MG922571	-
* Brycekendrickomyces acaciae *	CBS 124104	NG_058633	NR_132828	-
Byssochlamys lagunculariae ^*^	CBS 100.11	NG_058631	NR_144910	AY753354
* Capronia pilosella *	AFTOL-ID 657	DQ823099	DQ826737	-
Cladophialophora carrionii^*^	CBS 160.54	NG_055741	NR_121267	EU137201
* Exophiala aquamarina *	FMR 3998	KU705846	KU705829	-
E. aquamarina^*^	CBS 119918	-	NR_111626	JN112434
* E. brunnea *	CBS 587.66	MH870554	MH858890	JN112442
* E. equina *	CBS 116009	KF928497	KF928433	KF928561
E. equina ^*^	CBS 119.23	-	NR_111627	JN112462
E. jeanselmei^*^	CBS 507.90	MH873915	MH862234	EF551501
E. nigra^*^	CBS 535.94	NG_059253	NR_154974	-
* E. pisciphila *	AFTOL-ID 669	DQ823101	-	-
E. psychrophila^*^	CBS 191.87	MH873750	MH862061	JN112497
E. salmonis^*^	CBS 157.67	AY213702	NR_121270	JN112499
* E. xenobiotica *	CBS 115831	FJ358246	-	-
E. xenobiotica ^*^	CBS 118157	-	NR_111203	DQ182571
* Fonsecaea monophora *	CBS 102243	FJ358247	EU938579	EU938542
* F. pedrosoi *	BMU 07690	KJ930165	KJ701014	KM658155
F. pedrosoi ^*^	CBS 271.37	-	NR_130652	-
Marinophialophora garethjonesii^*^	MFLUCC 16–1449	-	NR_164246	-
Melanoctona tectonae^*^	MFLUCC 12–0389	KX258779	KX258778	-
* Metulocladosporiella musae *	CBS 113863	DQ008162	DQ008138	-
Paecilomyces fulvus ^*^	CBS 146.48	NG_063990	NR_103603	FJ389986
* Phialophora verrucosa *	BMU 07618	KJ930128	KJ700977	KM658080
P. verrucosa^*^	CBS 140325	-	NR_146242	-
* Rhinocladiella atrovirens *	CBS 317.33	MH866906	MH855447	-
Thysanorea lotorum^*^	CBS 235.78	MH872892	MH861130	-
T. papuana^*^	CBS 212.96	EU041871	EU041814	-
* T. rousseliana *	CBS 126086	MH875246	MH863784	-
* Veronaea aquatica *	JAUCC2549	MW046893	MW046892	MW248394
* V. botryosa *	CBS 102593	KF928493	KF928429	KF928557
* V. botryosa *	CBS 122236	KF928491	KF928427	KF928555
* V. botryosa *	MFLUCC 11–0072	MG922574	EU041817	-
V. botryosa ^*^	CBS 254.57	EU041873	EU041816	JN112505
V. compacta ^*^	CBS 268.75	EU041876	EU041818	-
* V. constricta *	CBS 572.90	MH873920	EU041819	-
* V. japonica *	CBS 776.84	NG_057789	EU041821	-
V. japonica ^*^	CBS 776.83	EU041875	EU041820	-
*Veronaea* sp.	DS253	-	MK808629	-
*Veronaea* sp.	E6917h	-	HM992819	-
*Veronaea* sp.	HB	-	KR909168	-
*Veronaea* sp.	NWHC 24266–02–03–03	-	KX148688	-
*Veronaea* sp.	NWHC 24266–02–04–01	-	KX148689	-

**Table 2.** Synopsis of related species

**Name**	**Conidiophore**	**Conidiogenous cell**	**Conidia**	**Conidial septation**	**References**
* Exophiala brunnea *	Branched 8-350 µm long	Occasionally intercalary, variable in shape, flask-shaped, ovoid, oblong, symmetrical or curved, fimbriate	Cylindrical to pyriform, proximally tapered and usually slightly stipitate 4.5-10 x 2-3 µm	aseptate	Papendorf (1969)
* Veronaea aquatica *	Loosely branched, sometimes geniculate, up to 280 × 2.4–4 µm	Occasionally intercalary, scars flat, rachis with crowded, flat to slightly	Cylindrical to pyriform, some subclavate, rounded at the apex 6.3–11(–11.8) × 2.4–3.7(–4.0) µm	0–1(–2)	This study
* Veronaea botryosa *	Unbranched 73–124.5 × 2–3 µm	Integrated, occasionally interspersed, flat to slightly prominent denticles, rachis with crowded	Ellipsoidal or fusiform, rounded at the apex (3–)6.5–8.5(–12) × (1.5–)2–2.5(–3) µm	1(–2)	Bonifaz et al. (2013), Dong et al. (2018)
* V. compacta *	Unbranched or branched at acute angles, rarely exceeding 50 µm	Occasionally intercalary, integrated, hardly prominent denticles, scars flat	Ellipsoidal to ovoid or oblong to subcylindrical, rounded at the apex, acropleurogenous (4–)6–7(–9) × 2–3 µm	0–1(–2)	Papendrof (1976)
* V. japonica *	Unbranched or occasionally branched 65 × 2–3 µm	Occasionally intercalary, hardly prominent denticles, scars flat, slightly pigmented	Ellipsoidal to ovoid, rounded at the apex (6–)7–8(–10) × 2–2.5(–4) µm	(0–)1	Arzanlou et al. (2007)
* V. oblongispora *	320 × 3–5 µm	Integrated, polyblastic, bearing thin, flat conidial scars	Oblong, obtuse at the apex 7–8 ×4–5 µm	aseptate	Morgan-Jones 1982
